# Recent advances in the management of acute ischemic stroke

**DOI:** 10.12688/f1000research.9191.1

**Published:** 2017-04-13

**Authors:** Philip Chang, Shyam Prabhakaran

**Affiliations:** 1 Northwestern University Feinberg School of Medicine, 303 East Chicago Avenue, Ward 12-140, Chicago, USA; 2 Northwestern University Feinberg School of Medicine, Abbott Hall Suite 1123, 710 N Lake Shore Drive, Chicago, USA

**Keywords:** Ischemic Stroke, Endovascular Therapy, Malignant Stroke, TeleStroke, Mobile Stroke Unit, ischemia, stroke

## Abstract

In recent years, several landmark trials have transformed acute ischemic stroke care. The most dramatic results from the field of acute endovascular intervention demonstrate unequivocal benefit for a select group of patients with moderate to severe deficits presenting within 7 hours from onset and with occlusions of proximal arteries in the anterior circulation. In addition, technological advances and workflow efficiencies have facilitated more rapid delivery of acute stroke interventions. This review provides an overview of recent advances in the management of acute ischemic stroke.

## Introduction

Until recently, recombinant tissue plasminogen activator (r-tPA) was the only acute ischemic stroke treatment. However, only 3–9% of patients with ischemic stroke actually receive r-tPA ^1– 4^, in part because of the limited time window for treatment. In 2008, the ECASS-3 trial extended the window for r-tPA eligibility from 3 to 4.5 hours after symptom onset, with additional exclusion criteria ^5^, which increased r-tPA utilization by as much as 20% in some centers ^6^. In 2015, the introduction of advanced endovascular treatment approaches further expanded this treatment window in select patients to up to 7 hours from symptom onset. Rapid assessment of suspected stroke patients to determine eligibility for these treatments, therefore, remains a critical step. Systems and processes to efficiently identify suspected stroke patients as early as possible and rapidly deliver acute reperfusion therapy are now commonplace. Further technological advances using telestroke and mobile stroke units in the prehospital setting have increased r-tPA utilization and reduced delays to treatment. This review will discuss the most recent developments in acute stroke treatment and improved systems for their delivery in current practice.

## Mechanisms and principles of acute ischemic stroke therapy

The most effective treatment for acute ischemic stroke is timely reperfusion of the causative vessel occlusion via r-tPA and/or mechanical thrombectomy. Reperfusion improves outcomes by reducing the volume of brain tissue injury. Therefore, a small core infarction (irreversibly lost brain tissue) with a large penumbra (viable but ischemic tissue) is the ideal target for acute reperfusion therapy. As time from initial stroke increases, the penumbra is quickly replaced by infarcted tissue ^7^. Therefore, swift reperfusion preserves tissue in the penumbra while reducing the size of the final infarct core, thereby limiting the volume of damaged tissue and reducing disability from stroke.

## Recent advances in acute endovascular therapy

Since 2015, there have been five prospective randomized clinical trials showing efficacy of endovascular thrombectomy in addition to standard management, typically r-tPA, in improving outcomes of acute ischemic stroke patients with proximal internal carotid artery (ICA) or middle cerebral artery (MCA) occlusions, moderate to severe stroke severity, and presenting within 12 hours of symptom onset ^8– 12^. Overall, there was over twofold increased odds of good outcome (independent of activities of daily living) with endovascular therapy compared to standard medical management with no increase in harm such as major intracranial bleeding. Because of these studies, the American Heart Association/American Stroke Association (AHA/ASA) released an update in 2015 ( Table 1) to their acute stroke management guidelines that recommended endovascular therapy for these select patients with acute ischemic stroke (class I, level of evidence A) ^13^.

**Table 1.** American Heart Association/American Stroke Association recommended selection criteria for mechanical thrombectomy in acute ischemic stroke ^14^.

Functionally independent pre-stroke (mRS score of 0 to 1)
Acute ischemic stroke receiving intravenous r-tPA within 4.5 hours of onset
Stroke caused by occlusion of the internal carotid artery or proximal (M1) middle cerebral artery on imaging (CT angiography)
Age ≥18 years old
NIHSS score of ≥6
CT brain without evidence of large infarct (ASPECTS score ≥6)
Treatment is able to be initiated (groin puncture) within 6 hours of symptom onset

ASPECTS, Alberta Stroke Program Early CT score; CT, computerized tomography; mRS, modified Rankin scale; NIHSS, National Institutes of Health stroke scale; r-tPA, recombinant tissue plasminogen activator.

Compared to prior failed trials, these studies had notable differences and advantages. First, the trials emphasized workflow efficiency with targets for door to groin puncture and reperfusion that were far more aggressive than in previous trials and therefore achieved times from symptom onset to reperfusion in less than 4 hours for many patients. Second, the use of stent-retrievers, superior to first-generation devices used in earlier trials, achieved greater rates of near-complete or complete reperfusion. Third, each trial included at minimum a vessel imaging screening test, typically computerized tomographic angiography (CTA), for the detection of large artery occlusion, avoiding the scenario encountered in prior trials wherein the target lesion was absent in a substantial number of patients enrolled, biasing the study towards a null result ^14^. In sum, these new trials validated long-held hypotheses that reperfusion is effective in patients with acute ischemic stroke due to large artery occlusion if provided early, when the majority of brain tissue is ischemic (reversible and salvageable) but not infarcted (irreversible and non-salvageable).

It is still unclear which imaging method is most appropriate in the acute evaluation of stroke patients for endovascular therapy. Two trials, MR-CLEAN and REVASCAT, utilized CTA and did not include any perfusion imaging prior to randomization ^8, 12^. The EXTEND-IA trial enrolled patients with CTA to confirm a proximal anterior circulation occlusion and CT perfusion imaging to confirm a small infarct core prior to randomization ^9^. The ESCAPE trial also used CTA scans to confirm a proximal anterior circulation occlusion but measured infarct burden using ASPECTS (Alberta Stroke Program Early CT score), a 10-point scoring system which evaluates 10 pre-determined areas of the brain supplied by the MCA. A point is deducted for hypodensities seen in each area on a standard CT scan, which included scores from 0 (imaging evidence of complete infarction of the MCA territory) to 10 (no imaging evidence of early infarction). Patients with small infarct burden (defined as an ASPECTS >5) were enrolled. Multi-phase CTA was used to assess collateral circulation. Patients who had good collateral circulation (filling of 50% or more of the MCA arterial circulation) were enrolled ^11^. SWIFT-PRIME utilized either MR angiography or CTA to identify proximal anterior circulation occlusion and the ASPECTS score to identify infarct core but included perfusion imaging in nearly 80% of patients enrolled using RAPID software ^15^ to automate the interpretation of penumbra and infarct core volumes ^10^. A meta-analysis of the major interventional trials showed that the benefit decreased every hour as time from stroke onset to time of arterial puncture for endovascular therapy, eventually becoming non-beneficial at 7.3 hours after stroke onset ^16^.

Currently, an initial CT scan without evidence of a large infarction by ASPECTS along with evidence of a proximal anterior circulation occlusion on CTA is the simplest approach to select patients for endovascular intervention, provided the procedure is initiated within 7 hours of stroke onset.

Though these trials ushered forth a new, exciting era for stroke intervention, further research is needed to evaluate whether more patients, such as those with basilar, vertebral, or posterior cerebral artery occlusions, may benefit from mechanical thrombectomy. In addition, treatment of occlusions beyond the first bifurcation of the MCA is uncertain, pending further study. Further research on optimal imaging selection, as will be discussed below, may be helpful in identifying patients who may benefit from intervention in delayed time windows.

## Expanding the treatment-eligible population

Only 25% of patients arrive at a hospital within 3.5 hours, and less than 65% arrive within 8 hours of symptom onset ^17^, excluding many patients from acute stroke reperfusion therapy. As a result, there is great interest in identifying a physiologic “tissue clock” rather than a chronological one (i.e. from last known well). Specific patient populations such as those who experience stroke symptoms upon awakening and mild stroke patients may benefit from reperfusion beyond the currently approved chronological timeline.

### Selecting wake-up stroke patients for r-tPA using MRI

About 15–25% of all ischemic strokes occur during sleep, without the patient knowing the exact time of symptom onset, often making the last known normal time greater than 6 hours before time of presentation ^18, 19^. As a result, these wake-up stroke (WUS) patients are excluded from r-tPA treatment and often mechanical thrombectomy as well.

The DEFUSE studies found early reperfusion was associated with improved clinical outcomes if there was a perfusion/diffusion mismatch (larger area of decreased perfusion than infarcted tissue) on MRI ^20, 21^. Other studies found that MR diffusion-weighted imaging (DWI) sequences identify acute ischemic changes within minutes of symptom onset while fluid attenuated inversion recovery (FLAIR) sequences identify changes 3–6 hours after symptom onset ^22^. Thus, a DWI-positive and FLAIR-negative MRI would suggest an acute ischemic stroke under 3 hours of symptom onset. The MR-WITNESS trial was a single-arm feasibility study which used the DWI/FLAIR selection criteria to administer r-tPA to 80 patients who woke up with stroke symptoms and presented to healthcare within 4.5 hours of waking up ^23^. Only one patient had a symptomatic hemorrhage, and the group had functional outcomes similar to those of the patients in the ECASS-3 trial, suggesting that this technique may be a safe and effective way of selecting more patients for r-tPA treatment. Currently, the WAKE-UP and EXTEND trials are underway to further investigate this approach in a larger cohort of patients with WUS ^24^. The DEFUSE III trial is a phase III trial investigating mechanical thrombectomy in patients with an ICA or proximal MCA occlusion and presenting between 6 and 16 hours from last known normal time, many of whom are WUS patients, using penumbra-core mismatch on perfusion imaging to identify stroke patients with salvageable tissue who could benefit from intervention ^25^.

## Decreased r-tPA dosage for populations with increased risk of intracranial hemorrhage

In the last few years, the Japanese drug safety authority approved r-tPA at a dosage of 0.6 mg/kg, a dose lower than the standard 0.9 mg/kg used for acute stroke treatment based on an uncontrolled open-label study done in 2006 ^26^. Since then, there is increasing concern that Asians in America may be at increased risk of intracranial hemorrhage after treatment of acute ischemic stroke with r-tPA ^27^. The recently published ENCHANTED trial investigated low-dose r-tPA (0.6 mg/kg versus 0.9 mg/kg) for patients with acute ischemic stroke and showed that there was a reduction in mortality and symptomatic intracerebral hemorrhage with a lower dosage when compared to the standard-dosage r-tPA, but this benefit was offset by an increased rate of disability at 90 days in the low-dose group. While the study failed to meet its primary endpoint, a secondary ordinal analysis of severity of disability based on the modified Rankin scale did show non-inferiority ^28^. While the lower dosage seems safer and may be suitable for particular high-risk populations, further research is needed to demonstrate efficacy.

## Improving stroke systems of care

### Delay in r-tPA administration leads to worse outcomes

Patients who received r-tPA within 90 minutes of symptom onset had better functional outcomes more than those who were treated at 181 to 270 minutes from onset ^29^. The number of patients needed to treat to achieve an outcome of functional independence increases from 3.5 patients in the first 90 minutes to 7 patients between 91 and 180 minutes and to 14 patients between 181 and 270 minutes ^30^. This is likely explained by the decreasing volume of viable brain tissue (e.g. penumbra) as time from symptom onset increases. Unfortunately, up to 69% of acute stroke patients are unable to get r-tPA secondary to delay in patient arrival ^31^, spurring initiatives to deliver stroke care as quickly as possible. Several proven options include expediting hospital arrival to intervention times, telestroke, and mobile stroke units.

### Improving door to needle (DTN) times

Target: Stroke is a quality initiative introduced by the AHA/ASA in 2010 which disseminated best practice strategies to achieve faster DTN times for r-tPA delivery at participating hospitals across the United States (US), with the goal of administering r-tPA in less than 60 minutes from hospital arrival in at least 50% of eligible patients. The best practices include 11 care strategies to achieve faster DTN: emergency medical services pre-notification to hospitals about stroke patients, hospital-specific stroke protocols, a rapid stroke identification and notification system, a single-call stroke activation system, direct to CT protocol, rapid transportation to imaging, rapid interpretation of imaging, rapid protocols for laboratory testing, pre-mixing of r-tPA in patients with high likelihood of stroke, a team-based approach, and a prompt feedback system. Implementation of these strategies resulted in a reduction of r-tPA administration times from a mean of 74 minutes to 59 minutes and reduced in-hospital mortality and long-term mortality ^32^. Because of this success, Target: Stroke Phase II is underway with an even more aggressive goal of administering r-tPA to at least 75% of patients within 60 minutes of arrival and at least 50% of patients within 45 minutes of arrival.

Internationally, a few DTN process improvement efforts have been even more stunning. An expedited protocol developed in Helsinki, Finland, in a single-provider area has demonstrated the most efficient DTN times in the world. Their ambulances have a direct line to a stroke neurologist, enabling the on-call neurologist to obtain a history and r-tPA consent over the phone as well as ready the stroke team at the hospital for prompt CT scanning and r-tPA administration before the patient arrives. The patient arrives and is taken from the ambulance to the CT scanner directly, during which time the neurologist performs a quick neurologic examination. After the CT scan excludes hemorrhage, r-tPA can be given immediately, often in the CT scanner, resulting in DTN times averaging 20 minutes ^33^. Three features of protocol – pre-notification by ambulances, direct-to-CT imaging, and pre-mixing r-tPA – were introduced in Melbourne, Australia, with similar results ^34^.

There are some limitations to applying the Helsinki protocol in the US. First, rather than one hospital in a region, most urban areas have multiple stroke centers. Close contact with ambulance systems and pre-notification of receiving hospitals may still help expedite r-tPA delivery but would require coordination across multiple hospital systems in a region. Second, the lack of universal health records in the US increases delays to treatment owing to time spent in eligibility determination. For example, use of anticoagulants and recent surgical history are relevant for r-tPA eligibility determination and require verification by patient or caregiver interview or medical record review. Third, a substantial portion of the US still does not reside within close proximity to designated stroke centers ^35^. In these instances, other solutions will be necessary.

### Telestroke

Telestroke permits remote audio-visual connection between a stroke center (the "hub") and multiple community hospitals (the "spokes"). Patients who arrive at non-stroke center hospitals, often smaller rural hospitals, may particularly benefit from telestroke, as these hospitals do not have access to an on-site stroke expert with experience in delivering r-tPA efficiently and safely ^36^. Studies have shown that telemedical consultation with a stroke neurologist remotely can reduce DTN times substantially at small hospitals ^37^. While the DTN times are higher than the Helsinki protocol, transport to the nearest hospital is faster than to the "hub" hospital, potentially resulting in similar onset-to-treatment times ^38^.

### Mobile stroke units

While DTN times are the focus of quality improvement at the hospital, reducing time from symptom onset to treatment makes the most impact on functional outcomes. Half of patients arrive at a hospital after 2 hours from symptom onset ^39^, and this has not changed over the last decade ^40^. The largest, and most important, window for reducing delays to treatment is therefore prior to hospital arrival. Programs have been developed to bring neurologists and r-tPA administration to the field in the form of a mobile stroke ambulance ^41^. These ambulances are equipped with a mobile CT scanner and point-of-care laboratory testing ^42^. In some programs, a vascular neurologist travels in the rig for in-field interview and examination before administration of r-tPA ^43^. Other programs have installed telemedicine equipment for remote interview and examination by a neurologist with the aid of emergency medical technicians in the ambulance ^44^.

The prospective PHANTOM-S study compared conventional care to pre-hospital thrombolysis with r-tPA with an in-field emergency-trained vascular neurologist. Significantly more patients received r-tPA in the pre-hospital thrombolysis arm of the study versus conventional care (31% versus 4.9%, *P*<0.001). Among patients who received r-tPA, the pre-hospital thrombolysis arm had a remarkably lower median onset-to-intervention time (50 minutes versus 105 minutes, *P*<0.001) ^45^. In a comparative analysis of symptom onset to r-tPA treatment times, pre-hospital thrombolysis with a mobile stroke unit provides the best times, even when compared to the Helsinki protocol and telestroke programs ^46^. However, this comes at a very large financial cost (e.g. initial outfitting of the ambulance and subsequent maintenance), which may be an obstacle for implementation within many healthcare systems ^47^.

## Conclusions

With the technological advances in acute stroke care, many more patients with the most severe strokes can receive treatments that can reduce death and disability from the disease. Endovascular intervention with mechanical thrombectomy has been shown to improve functional outcomes in select patients with severe stroke and large artery occlusion in the anterior cerebral circulation. Administration of r-tPA has been shown to be effective up to 4.5 hours from symptom onset. New protocols have been developed to potentially expand r-tPA treatment to patients with WUS, approximately 15–25% of all ischemic strokes and mild stroke, and up to 75% of all ischemic strokes. Advances in technology have also led to models of care to deliver r-tPA in a speedy and efficient manner, whether through pre-hospital triage via telephone, r-tPA administration via telemedicine, or actually administering r-tPA in the ambulance. These innovations have revolutionized acute ischemic stroke care from a disease with no treatments to one with multiple proven options.
